# Genetics and Genomics of Chronic Pancreatitis with a Focus on Disease Biology and Molecular Pathogenesis

**DOI:** 10.1055/s-0043-1776981

**Published:** 2023-11-22

**Authors:** Erum Khan, Soura Chakrabarty, Sanobar Shariff, Mainak Bardhan

**Affiliations:** 1 Department of Neurology, Alzheimer's Disease Research Center, The university of Alabama at Birmingham, Birmingham, United States; 2 Department of Pathology, University of Cambridge, Cambridge, United Kingdom; 3 Yerevan State Medical University, Yerevan, Armenia; 4 Department of Medical Oncology, Miami Cancer Institute, Baptist Health South Florida, Miami, Florida, United States

**Keywords:** pancreatitis, chronic pancreatitis, genetics, genomics

## Abstract

Chronic pancreatitis is a long-term fibroinflammatory condition of the pancreas with varying incidences across countries. The recent increase in its occurrence implies the involvement of genetic, hereditary, and unconventional risk factors. However, there is a lack of updated literature on recent advances in genetic polymorphisms of chronic pancreatitis. Therefore, this review aims to present recent findings on the genetic implications of chronic pancreatitis based on individual gene mechanisms and to discuss epigenetics and epistasis involved in the disease. Four mechanisms have been implicated in the pathogenesis of chronic pancreatitis, including premature activation of proteases, endoplasmic reticulum stress, ductal pathway dysfunction, and inflammatory pathway dysfunction. These mechanisms involve genes such as *PRSS1, PRSS2, SPINK, CEL, PNLIP, PNLIPRP2, CFTR, CaSR, CLDN2, Alpha 1 antitrypsin, and GGT1* . Studying genetic polymorphisms on the basis of altered genes and their products may aid clinicians in identifying predispositions in patients with and without common risk factors. Further research may also identify associations between genetic predispositions and disease staging or prognosis, leading to personalized treatment protocols and precision medicine.

## Introduction

Chronic pancreatitis (CP) is a long-lasting recurring or continuous inflammation of the pancreas. It is characterized by fibrosing changes in the pancreas with partial to complete loss of endocrine and exocrine functions. The annual incidence of CP is about 50/100,000 persons with a wide variation, especially in tropical countries like India (20–125 per 100,000). The mortality is around 1 to 7% in patients and increases to 10 to 18% in patients with severe pancreatitis, which involves infection and necrosis leading to organ failure. 1 Genome-wide association studies (GWAS) and family studies have identified many genes implicated in the earlier classified, idiopathic CP. This review aims to acknowledge the genetic polymorphisms in CP, which may or may not interact with secondary and metabolic causes.

### Epidemiology

CP is known to affect 30 to 100 per 100,000 individuals and is estimated to be higher since the epidemiological data reported from hospitals and national records are subjected to hurdles in patient compliance, record management, etc. 2 The male-to-female ratio ranges from 1.05:1 to 5:1. CP has a strong association with alcoholism, with an incidence of 3.5 to 15 per 100,000 individuals. 3 . However, alcoholism being a lower risk factor for women and children, it is postulated that CP might be due to genetic disorders or polymorphisms, autoimmune or hereditary patterns of diseases, congenital anomalies, trauma, or other toxins. 3 4 5

### Chronic Pancreatitis: Clinical Characteristics

In CP, mild edematous to severe necrotizing inflammation occurs, which shows irregular sclerosis focally or diffusely in the parenchyma and abnormal dilation, strictures, or plugs in the ductular system. Clinically, it presents with epigastric pain associated with nausea, vomiting, and anorexia, which characteristically but rarely radiates to the back, forcing the patient in a bend-over back position. Weight loss with bulky and greasy stool and insulin-dependent diabetes mellitus are frequent consequences and common complications. These also include compression of adjacent structures and eventual infection, calcification, necrosis, and rupture of pseudocysts. Sometimes, the condition may progress to malignancy with risk as high as 50 to 60 times than the general population. 6

### Risk Factors

Risk factors include alcohol, toxins, smoking, trauma, etc.., as included in the toxic-metabolic, idiopathic, genetic, autoimmune, recurrent and severe acute pancreatitis and obstructive (TIGAR-O) pancreatitis risk/etiology checklist. 7

Alcohol has been implicated not just as a risk factor but also in the alteration of the genome predisposing to CP through genetic polymorphisms. 4 8 9 10 Similar associations have been found with smoking. 11 12 13 Several congenital anomalies with genetic predispositions can also be considered important predisposing factors. 14 15 The TIGARO checklist also contains metabolic factors like hypercalcemia, 16 hypertriglyceridemia, 17 malignancy, kidney diseases, oxidative stress factors, and other toxins. 18 19 Many authors also advocate consideration of diabetes mellitus, obesity, Wilson's disease, glycogen storage disorders such as Von Gierke's disease, and the genes implicated therein as risk factors for CP. 16 20 21

Nonetheless, after thorough investigations of the above-mentioned factors, causally implicated genotypes, and ruling out exotic etiologies like tropical pancreatitis endemic to South India, some patients are diagnosed with idiopathic CP.

## Genetics Chronic Pancreatitis

### Pathological Mechanisms

The genes implicated in CP can be attributed to four different pathological mechanisms. First, the premature activation of trypsinogen and other proteolytic enzymes directly destroys the parenchyma and ductular system, followed by the accumulation of misfolded proteins and their by-products that induce endoplasmic reticulum (ER) stress, which further activates the inflammatory cascade. The third pertains to ductular dysfunction, which leads to their maturation and activation when they are in the vicinity of the parenchyma and have the potential to destroy it. The fourth mechanism involves the activation of mediators of inflammatory pathways due to direct genetic polymorphisms or transcription factor mutations. Several epigenetic factors have also linked the risk factors, as mentioned previously in the TIGARO classification, with potential genetic predispositions.

### Mechanism A: Premature Activation of Proteases

The *PRSS1* and *PRSS2* genes control trypsinogen and cathepsin L to activate and decrease the degradation of trypsinogen. The *SPINK* gene inhibits trypsin activation. *CTRC* and *CTRB* control cathepsin B and *CTRC* , which regulate trypsinogen activation. The trypsinogen activation further controls chymotrypsinogen, proelastase, and procarboxypeptidase activation.

#### *PRSS1* and *PRSS2* Gene

Cationic trypsinogen in humans is 3.6 kb long and separated into five exons on chromosome 7. It is embedded within the human T-cell receptor. Cationic and anionic trypsinogen genes are positioned in 3 inches end of the same chromosome; however, the mesotrypsinogen encoding gene locus is on the short arm of chromosome 9.

We observe activation of cationic trypsinogen degradation, enhancing CTRC-mediated processing of activation peptide, direct stimulation of activation, and reduction of trypsinogen activation as most common ones. 22 Rare mutations include p.D19A, p.D21A, p.D22G, p.K23R, and p.K23_I24insIDK, which enhance autoactivation and −28 delTCC, which increases the level of trypsinogen transcription. 23 24 25 26

In summary, *PRSS1* mutations increase cationic trypsinogen activation by lowering *CTRC* -dependent trypsinogen degradation, boosting *CTRC* -mediated activation peptide processing, or directly promoting autoactivation.

The *PRSS* mutations also have a wide spectrum of clinical presentation, with A16V mutation being the mildest variety. 27 Hence as pointed out by Witt et al, 32 mutations can lead to variable clinical outcomes, and care should be taken in reporting and interpreting rare or novel mutations.

Table 1 details the discovery of *PRSS* as a genetic implication in CP, its pathology, mutations, and mode of inheritance.

**Table 1:** Discovery, pathophysiology, and inheritance of common mutations in *PRSS 1* , *PRSS2* , *SPINK1* , and *CTRC* genes

Gene	Discovery of its implication in chronic pancreatitis (CP)	Mutation	Pathophysiology	Inheritance
*PRSS 1* p.R122H > p.N29I > p.A16V ∼ p.R122C > p.N29T > p.V39A	1996 1	p.N29I 2	Markedly increases trypsinogen autoactivation	Autosomal dominant hereditary pancreatitis 71
		p.R122C, p.R122H 3	Prevent CTRC-mediated trypsinogen degradation	Autosomal dominant hereditary pancreatitis
		p.A16V 4	Enhances autoactivation	Autosomal dominant hereditary pancreatitis (much less penetrance)
		p.P17T 5	Enhances autoactivation	Autosomal dominant hereditary pancreatitis
		c. − 204C > A 6 7	Reduces trypsinogen expression with more pronounced effect on alcoholic CP	Autosomal dominant hereditary pancreatitis
*PRSS2*	2006 8 9	p.G191R	Increases autocatalytic proteolysis and inactivation	–
*SPINK1*	2000 10	p.N34S	Decreased expression of serine protease inhibitor Kazal type 1	Autosomal recessive with low penetrance 11
		c.194 + 2T > C (sometimes with c. − 215G > A, which may sometimes mitigate its effect) 10 12 13	Diminishes SPINK expression 14	Autosomal dominant inheritance with variable penetrance
		IVS3 + 2T > C 10 15	Splicing aberrations inhibit SPINK expression	Autosomal dominant inheritance with variable penetrance
		M1T	Knocks out start codon to stop the product formation altogether	Autosomal dominant inheritance
*CTRC*	2008 16	p.A73T	Secretion defect	–
		p.K247_R254del 17	Inactive protein formed, which is also prone to degradation	–
		p.R254W	Degraded by trypsin	–
		p.V235I	Partially reduced activity	–
		p.G60 18 19	Altered pre-mRNA splicing, which reduces mRNA expression	–
		Inversion at the *CTRB1/CTRB2* locus 20 21	The CTRB1-to-CTRB2 ratio is altered, which reduces protective trypsinogen degradation	–

#### *SPINK1* Mutations

Since its discovery in 1948, *SPINK/PSTI* (pancreatic secretory trypsin inhibitor)/ *TATI* (tumor-associated trypsin inhibitor) from bovine pancreas has been found in all animal species and several human tissues, including the kidney, liver, ovary, and breast too, apart from the pancreas—implying its functions as an acute phase reactant protein, protector of GIT mucus layer, and helper in repair after injury. 26 It forms a covalent bond restricting the activation of trypsin temporarily, after which trypsin regains autoactivation.

Surprisingly, acinar cells do not express *SPINK1* , making it a primary risk factor for developing recurrent acute pancreatitis (RAP) or CP. This suggests that it represents a failed feedback inhibition of recurrent trypsin activation. 27 28

N34S mutation in the past has been associated with protease/trypsin pathology and ER premature degradation pathophysiology like *CFTR* and *Alpha 1 antitrypsin genes* . 29 30 However, this will require one of the intronic variants (IVS1-37 T > C, IVS2 + 268 A > G, IVS3-604 G > A, and IVS3-66_-65 insTTTT), which are in linkage disequilibrium with N34S. Hence, authors recently have started including it under the former pathology. 31

Some rare mutations like IVS3 þ 2 T > C, L14P, D50S, IVS3 þ 184 T > A, IVS3 þ 125 C > A, and c.27delC are also reported, but their pathology is indeterminant. 32

Table 1 details the discovery of *SPINK* as a genetic implication in CP, its pathology, mutations, and mode of inheritance.

#### *CTRC* Mutations

The *CTRC* gene located on chromosome 1 spans 8.2 kb. A tryptic cleavage of Arg-Val peptide bond at the propeptide C-terminal end results in the enzyme's activation. 33 34

The mutations that cause loss of the *CTRC* function include mechanisms of misfolding, resistance to trypsin-mediated activation, deficiency of catalytic activity, and increased degradation. 35 The polymorphic variants also showed statistical significance in alcohol-related CP and tropical pancreatitis, making it contribute to secondary CP as well. 36 37

**Fig. 1:**
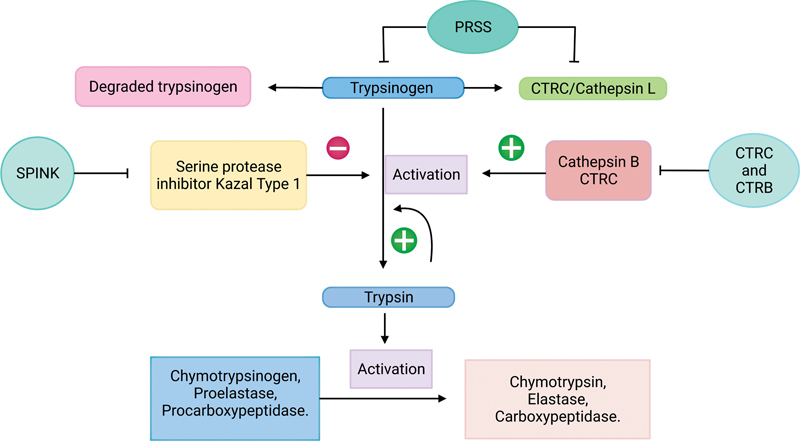
Pathway and genes involved in premature activation of proteases.

Table 1 details the discovery of *CTRC* as a genetic implication in CP, its pathology, mutations, and mode of inheritance, and Fig. 1 gives a schematic representation of mechanism A.

### Mechanism B: Predisposition to ER Stress

The degraded trypsin, other digestive enzymes, carboxyl esters, pancreatic lipase, and carboxypeptidase 1 may accumulate in the cell, predisposing it to ER stress. Thus, the genes regulating them are implicated in CP, that is, *PRSS, CEL, PNLIP, PNLIPRP2* , and *CPA1.*

#### *PRSS1*

Kereszturi et al defined misfolding phenotypes of digestive enzyme mutants. These give rise to proteins secreted poorly from transfected cells but are usually detectable in cell lysates as protease-sensitive forms. The final common pathway leading to CP remains the same for *PRSS* mutations, and mutations of digestive enzyme accumulation, nonsecretion, or excessive degradation (much more than the former two compared to *PRSS* mutations) cause ER stress, apoptosis, and tissue inflammation. 25

Table 2 details the discovery of *PRSS* as a genetic implication in CP, its pathology with respect to ER stress induction, mutations, and mode of inheritance.

**Table 2:** Discovery, pathophysiology and inheritance of common mutations in *PRSS1, CPA1* , *CEL* , *PNLIP* , and *PNLIPRP2* genes leading to endoplasmic reticulum (ER) stress

Gene	Discovery of its implication in chronic pancreatitis	Mutation	Pathophysiology	Inheritance
*PRSS1*	2009 22	p.R116C	Intracellular retention of misfolded protein and ER stress	Hereditary and sporadic 23
		p.C139S	Intracellular retention of misfolded protein and ER stress	Sporadic 23
		p.L104P	Intracellular retention of misfolded protein and ER stress	–
		p.D100H 24	Misfolding predisposing to apoptosis without increasing ER stress	Autosomal recessive pattern of inheritance 23
		p.C139F 25	Misfolding predisposing to apoptosis without increasing ER stress	–
*CPA1*	2013	p.N256K	Impaired catalysis, reduced secretion or degradation by the activating proteases, which reduces the “apparent activity” of the gene product	–
		p.R382W	Impaired catalysis, reduced secretion or degradation by the activating proteases, which reduces the “apparent activity” of the gene product	–
		c.1073-2A > G	Impaired catalysis, reduced secretion or degradation by the activating proteases, which reduces the “apparent activity” of the gene product	–
		p.S282P	Misfolding that induced ER stress	Autosomal dominant hereditary pancreatitis
*CEL*	2006 26	c.1785delC and c.1686delT	Aggregation prone CEL lipases lead to exocrine dysfunction and acinar loss 27	Autosomal dominant
		*CEL–HYB1*	Hybrid protein is misfolded and secreted poorly due to retention precipitating ER stress 28	–
*PNLIP*	2015	p.T221M 29	Intracellular retention and diminished secretion associated with ER stress	Autosomal recessive
*PNLIPRP2*	2011	p.W358X 30	Misfolding-induced ER stress	Autosomal recessive

#### *CPA1* Gene

Till as recently as 2013, misfolded digestive enzyme studies were focused on trypsin, when evidence of involvement of the *CPA 1* gene was provided. 39 CPA is a digestive zinc-dependent carboxypeptidase that assists in dietary polypeptide digestion. *CPA1* and *CPA2* are A-type carboxypeptidases that act on aromatic and aliphatic amino acid residues exposed by chymotrypsins and elastases. In contrast, type B carboxypeptidase ( *CPB1* ) hydrolyzes C-terminal Lys and Arg residues produced by tryptic digests.

Its gene is located on the seventh chromosome, spanning 8 kb and containing 10 exons. The preprotein, made up of 419 amino acids, is activated by catalyzation through trypsin and *CTRC* .

Table 2 details the discovery of *CTRC* as a genetic implication in CP, its pathology, mutations, and mode of inheritance.

#### *CEL* Gene

The *CEL* locus on chromosome 9 includes *CELP* , which differs from *CEL* by an exon 2-7 of *CEL* . The *CEL* gene encodes for carboxyl ester lipase, is involved in lipid and cholesterol digestion, and is activated by bile salts in the duodenum. 40 41

Previously reported single-base deletions in *CEL VNTR* causing MODY type 8 characterized by exocrine insufficiency led scientists to think in the direction of it being a possible risk effector for CP. 42

*CEL-HYB* encodes a CEL protein with a functional *CEL* enzyme region but a shorter and different *VNTR* contributed by the *CEL* pseudogene. However, confusion revolves around its pathophysiology, causing CP, with some authors identifying the new protein C terminus and not the lack of normal *VNTR* to cause lack of normal secretion of the *CEL* gene. 43 Nonetheless, there is consensus that increased lipase activity is unrelated to disease pathogenesis.

Table 2 details the discovery of *CEL* as a genetic implication in CP, its pathology, mutations, and mode of inheritance.

#### *PNLIP* and *PNLIPRP2* Gene

*PNLIP* and *PNLIPRP2* are part of a gene cluster encoding *PTL* , *PLRP1* , and *PLRP2* on chromosome 10. It forms a 56-kDa protein essential for hydrolysis and absorption of long-chain fatty acids with the help of its cofactor, CLPS. Since very few patients have been identified with this mutation as a causative factor of hereditary CP, the literature lacks more insights into its pathophysiology.

**Fig. 2:**
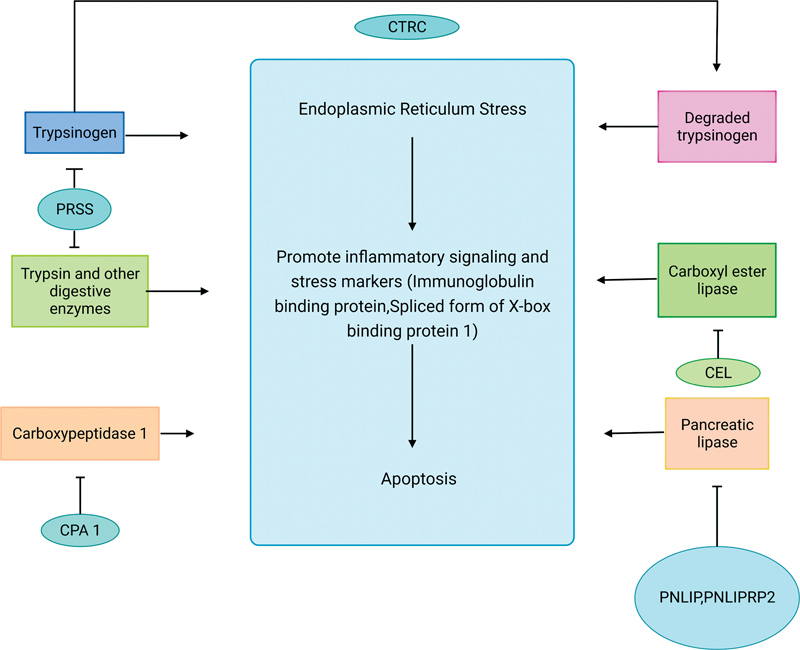
Mechanism involved in predisposition to endoplasmic reticulum (ER) stress.

Table 2 details the discovery of *PNLIP* as a genetic implication in CP, its pathology, mutations, and mode of inheritance, and Fig. 2 provides a schematic representation of mechanism B.

### Mechanism C: CP through the Ductal Pathway Dysfunction

The ductal function involves selective secretion, absorption, and prevention of unwanted leakage regulated by *CFTR* , *CaSR* , and *CLDN2* genes. Ineffective pancreatic juice secretion would adversely harm the ductular and, subsequently, the parenchymal region, predisposing to CP.

- *CFTR* -R117H, L237R, 5T Allele, R75Q found with *CFTR* mutations: *CFTR* is located on the seventh chromosome and encodes a transmembrane protein present on the surface of epithelial cells and in response to cyclic AMP responsive chloride channel on most epithelial cells. Its absence from the acinar cells makes it unique to the ductal pathway dysfunction pathophysiology of CP. A decreased pH of ductal and acinar lumen leads to coagulation or solubilization of proteins, which may contribute by damaging the epithelial barrier or enhanced autoactivation of trypsinogen since that is also highly dependent on pH.
Class I mutations result in improper mRNA transcription, class II mutations result in defective protein processing, and class III mutations result in little or no functional protein and are frequently associated with a severe outcome. Reduced conductance (class IV) or protein quantity (class V) mutations decrease but do not abolish *CFTR* function and are frequently associated with a moderate phenotype. 44
  Table 3 details the discovery of *CFTR* as a genetic implication in CP, its pathology, mutations, and mode of inheritance.
- ***CLDN2*gene:***CLDN2* is a tight junction protein that forms low resistance, cation-selective ion, and water channels between endothelial cells, and its expression is increased during stress or damage where it can be expressed on acinar cells. 33 45 The *MORC4* protein is a transcription factor; hence, being near to *CLDN2* and contributing to its regulation implicates it being a potential risk for CP. Nonetheless, pancreatitis is not correlated with its expression.
  Table 3 details the discovery of CLDN2 as a genetic implication in CP, its pathology, mutations, and mode of inheritance.

**Table 3:** Discovery, pathophysiology, and inheritance of common mutations in *CFTR* , *CLDN2* , and *CASR* genes

Gene	Discovery of its implication in chronic pancreatitis	Mutation	Pathophysiology	Inheritance
*CFTR*	1998 31	p.F508del mutation	Pancreatic insufficiency leading to exocrine and endocrine insufficiency	Autosomal recessive
		p.R117H	Pancreatic insufficiency leading to exocrine and endocrine insufficiency	Autosomal recessive
		R75Q	Pancreatic insufficiency leading to exocrine and endocrine insufficiency specifically affecting the bicarbonate section and not the chloride section of the channel	Autosomal recessive
CLDN2–MORC4 locus	2013 32	CLDN2 (rs4409525, rs12008279) MORC4 (rs12688220, rs6622126)	Atypical localization disrupting the tight junctions	X linked
*CASR*	2003 33	p.L173P, p.V477A, p.A986S, p.Q1011	Electrolyte imbalance in the lumen directly damages and triggers trypsinogen autoactivation	Autosomal dominant
		p.A986S and p.Q1011E	Increase serum calcium levels	
		p.R990G	Facilitates fibrosis usually in the setting of alcohol	

#### CaSR Gene

*CaSR* is a plasma membrane bound G protein coupled receptor (GPCR) for extracellular calcium level modulation that is expressed in parathyroid, bone, kidney, and brain, with the exception of pancreatic ducts and acini.

Its dysfunction implicated in increased calcium levels and linkage with increase of cAMP and bicarbonate secretion are the potential mechanisms by which it can increase the risk for CP. 46

Hence, *CaSR* controls electrolyte and fluid secretion and functions as a sensor and regulator of pancreatic juice calcium content and bicarbonate secretion. When its levels are elevated by washing out duct fluid with higher Ca levels, it would trigger trypsinogen autoactivation and also its stabilization. 47

**Fig. 3:**
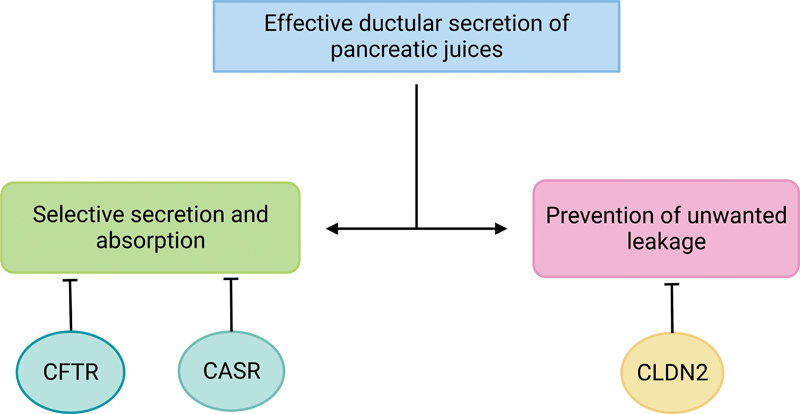
Chronic pancreatitis (CP) through the ductal pathway dysfunction.

Table 3 details the discovery of *CaSR* as genetic implication in CP, its pathology, mutations, and mode of inheritance. Fig. 3 gives a schematic representation of mechanism C.

### Mechanism D: CP through the Inflammatory Pathway Dysfunction

Several genes involved in inflammatory cascade or contributing to trigger or diffuse it have been implicated in CP as it is a chronic inflammatory pathology characterized by exocrine dysfunction with or without endocrine dysfunction and fibrosis.

#### Alpha 1 Antitrypsin Gene

It generates serum inhibitors of proteolytic enzymes such as neutrophil elastase, cathepsin G, proteinase 3, and trypsin, and is 12.2 kB long. This gene is located on the long arm of chromosome 14. Genetic defects commonly found leading to gene deficiency are *E264V* (PiS) and *E342K* (PiZ). The PiZ allele was initially discovered to be significantly more common in CP than controls in a South African study in 1975. 48 Many authors have also found significant associations of lesser alpha 1 antitrypsin with alcohol-induced CP. 49 50

The pathophysiology remains straightforward with decreased inactivation of trypsinogen, but it is hypothesized that heterozygous states may also predispose to CP by accelerating the damage done by typical triggers like alcohol and hypercalcemic states.

#### *GGT*

*GGT1* encodes for γ-glutamyl transpeptidase, an extracellular enzyme expressed in the epithelium of ducts and tubules of the kidney, epididymis, prostate, gallbladder, and biliary tree, with its chief function to salvage the amino acids glycine, glutamate, and cysteine from glutathione. It provides cysteine for the rate-limiting phase of glutathione production by hydrolyzing reduced glutathione's gamma carboxyl amine bond. Intracellular glutathione protects cells from oxidative stress, controls redox signaling, and aids in detoxification. Cell proliferation, apoptosis, immunity, and fibrosis are also controlled by the same. 51

Single nucleotide polymorphisms like *rs4820599* , *rs8135987* , and *rs2017869* have been implicated in hereditary CP as part of a major haplotype. 33

*GGT1 rs5751901* has also been involved in having lower *GGT* values, particularly in smokers with CP and AP. 52 53 54

*rs202087650* ( *p.S347S* found in an altered HNF4 binding motif) and *rs569846079* (intronic) are risk factors that modify transcription factors marked by histone modifications. 33

#### Tumor Necrosis Factor Alpha

Immunostimulatory and proinflammatory qualities have been found in tumor necrosis factor alpha (TNF-alpha) since 1992 by increasing the expression of adhesion molecules and neutrophil activation, co-stimulation of T-cell activation, and B-cell antibody production. 55

Its mutation, –308A/G polymorphism in the promotor region, elevates the expression of TNF-alpha. Nonetheless, authors like Yang et al 56 and Liu et al 57 have denied these associations in their meta-analyses.

According to researchers, TNF-alpha's function to activate stellate cells is thought to play a crucial role in the pathophysiology of pancreatitis, leading to persistent inflammatory cell infiltration, acinar cell degeneration, and fibrosis. 58 59

Apart from the genes mentioned earlier, *p16(INK4a)* , glutathione S transferase, and matrix metalloproteinases have been implicated in the inflammatory pathophysiology of CP, but whether their involvement is a cause or effect of the generalized inflammatory response seen in CP needs further clarification. 60

## Epigenetics and Epistasis

The speculations of some environmental and genetic interaction in the etiology of CP stemmed from the similarity in the histology, which suggested that a common intersection of the pathological pathway exists. 61

Statistical inferences can be robust if the clinical studies with high power are large in number, but that is difficult in genetic causes of CP where the variance is low, and the factor being estimated has a key effect. Nonetheless, recent studies and meta-analysis have identified some interactions of genes within themselves and with the environment, which we highlight below.

Since Noone et al 63 raised the hypothesis of *SPINK1* ( *N345S* ) interaction with *CFTR* genotypes (F508del), several large cohort studies like the Pittsburgh hereditary pancreatitis study were conducted, and meta-analysis from their reports showed *CFTR* severe (functional classes 1 to 3) and *CFTR* mild (functional class 4) were associated with *SPINK1* mutation in pancreatitis cases. 63

When numerous *SPINK/CFTR* variations are present, the risk is synergistic mutation specific, and healthy carriers appear to be rare. Hence, it is in congruence with the hypothesis of multiple genetic interactions among themselves intersecting at a common pathological pathway.

The environmental interaction hypothesis has invariably involved alcohol as one of the primary suspects, but with the difficulty of finding healthy alcoholic controls, small and limited studies are available to infer from. 64

Cytochrome P450 2E1 metabolizes low-molecular-weight compounds, including drugs, toxins, and other substrates. 65 66 Its 5′ flanking region ( *Rsal/Pstl* ) and 6′ flanking region ( *Dral* ) point mutations have been reported to affect the susceptibility of alcoholics to CP. 67 68

CYP2E1 plays a crucial role in the initial stage of alcohol metabolism in the microsomal ethanol oxidizing system. 60

Similarly, *ADH2* and *ADH3* are polymorphic genes that code for distinct types of subunits with differing characteristics. The three alleles are ADH2*1 , ADH2*2 (usually found in white and yellow races), and ADH2*3 (found in Afro-American races), responsible for b1, b2, and b3 subunits, which unite together to form heterodimers and homodimers. Since the first hypothesis of implications of *ADH* gene in CP through alcohol in the pathology in 1975, 69 many studies have been conducted whose meta-analyses have identified both protective and causative genes in the CP. The ADH3*1 and ADH3*2 homozygotes and heterozygotes seem conducive to CP, 70 with ADH3*1 having stronger evidence than the ADH3*2 alleles. On the other hand, some studies suggest that the ADH2*2 allele may protect against this condition in a portion of the population, whereas others disagree. CP has a degree of co-occurrence in duct cell ( *CFTR, CPA1,* and *PRSS1* ) genes and stress response genes ( *GGT1* gene). Studies with higher power would further bring out these occurrences significantly. This, nonetheless, suggests that in patients lacking highly penetrant pathogenic mutations like *CF* and *PRSS1* , hereditary pancreatitis, both acinar and ductal cell types, may be required to cause pancreatitis.

## Conclusion

Literature is brimming with information about risk factors, genes implicated, pathophysiology, and predispositions of CP. Recent advances have also evolved diagnostic and imaging techniques for the same. Therapeutic, medicinal, and surgical procedures, including interventional endoscopy and surgery, can effectively provide long-lasting symptomatic treatment. However, CP care needs staging, characterization, and prognostic markers for monitoring the disease. This study details the knowledge gained in identifying genetic elements in CP and hence calls for more detailed studies concerning genetics and clinical stratification. These clinical trials and meta-analyses will further guide therapeutic strategies and pave the way for precision medicine.
